# 3β-Hydroxysterol Δ24-Reductase on the Surface of Hepatitis C Virus-Related Hepatocellular Carcinoma Cells Can Be a Target for Molecular Targeting Therapy

**DOI:** 10.1371/journal.pone.0124197

**Published:** 2015-04-13

**Authors:** Makoto Saito, Takashi Takano, Tomohiro Nishimura, Michinori Kohara, Kyoko Tsukiyama-Kohara

**Affiliations:** 1 Department of Experimental Phylaxiology, Faculty of Life Sciences, Kumamoto University, 1-1-1 Honjo Kumamoto-City, Kumamoto, Japan; 2 Department of Microbiology and Cell Biology, Tokyo Metropolitan Institute of Medical Science, 2-1-6 Kamikitazawa, Setagaya-ku, Tokyo, Japan; 3 Division of Veterinary Public Health, Nippon Veterinary and Life Science University, 1-7-1 Kyonan, Musashino, Tokyo, Japan; 4 Chemo-Sero-Therapeutic Research Institute, Kikuchi Research Center, Kyokushi, Kikuchi, Kumamoto, Japan; 5 Transboundary Animal Diseases Center, Joint Faculty of Veterinary Medicine, Kagoshima University, Kagoshima, 1-21-24 Korimoto, Kagoshima, Japan; 6 Laboratory of Animal Hygiene, Joint Faculty of Veterinary Medicine, Kagoshima University, Kagoshima, 1-21-24 Korimoto, Kagoshima, Japan; Saint Louis University, UNITED STATES

## Abstract

In our previous study, we demonstrated that 3β-hydroxysterol Δ24-reductase (DHCR24) was overexpressed in hepatitis C virus (HCV)-related hepatocellular carcinoma (HCC), and that its expression was induced by HCV. Using a monoclonal antibody against DHCR24 (2-152a MAb), we found that DHCR24 was specifically expressed on the surface of HCC cell lines. Based on these findings, we aimed to establish a novel targeting strategy using 2-152a MAb to treat HCV-related HCC. In the present study, we examined the antitumor activity of 2-152a MAb. In the presence of complement, HCC-derived HuH-7 cells were killed by treatment with 2-152a MAb, which was mediated by complement-dependent cytotoxicity (CDC). In addition, the antigen recognition domain of 2-152a MAb was responsible for the unique anti-HCV activity. These findings demonstrate the feasibility of using 2-152a MAb for antibody therapy against HCV-related HCC. In addition, surface DHCR24 on HCC cells exhibited a functional property, agonist-induced internalization. We showed that 2-152a MAb-mediated binding of a cytotoxic agent (a saponin-conjugated secondary antibody) to surface DHCR24 led to significant cytotoxicity. This suggests that surface DHCR24 on HCC cells can function as a carrier for internalization. Therefore, surface DHCR24 could be a valuable target for HCV-related HCC therapy, and 2-152a MAb appears to be useful for this targeted therapy.

## Introduction

Hepatocellular carcinoma (HCC) is the fifth most common cancer and the third most common cause of cancer death worldwide [1]. It is also the major cause of death in patients with chronic hepatitis C virus (HCV) infection [2]. Accumulating epidemiological evidence has shown that persistent infection with HCV is a major risk factor for the development of HCC [3]. Once chronic HCV infection develops into cirrhosis and ultimately progresses to HCC, a radical cure is very difficult to achieve through replication suppression and elimination of HCV using antiviral drugs and interferon. In such cases, chemotherapy and surgical resection are inevitable. However, with chemotherapy (anticancer drugs), harmful side effects are a concern due to their considerable impact on drug metabolism, which is related to the deteriorated liver function of HCC patients. In addition, the tumor response rate of HCC patients receiving systemic chemotherapy is low, and chemoresistance can easily develop [4]. Current therapeutic agents, including interferon and anticancer drugs, have side effects because they do not specifically act on the infected cells and cancer cells. In addition, the use of surgical resection is limited to early stage HCC. At present, liver transplantation is the most effective therapeutic approach for liver dysfunction due to progression from chronic hepatitis to cirrhosis and HCC; however, hepatitis frequently recurs after transplantation in patients with hepatitis C [5,6]. Therefore, additional effective treatments are needed for HCV-related HCC that have the potential to not only specifically kill cancer cells but also eliminate HCV.

In recent years, emerging insights into the biology and molecular signaling pathways of cancer cells have led to the identification of potential targets and promising targeted therapies for the treatment of HCC. Sorafenib, a multi-kinase inhibitor, is a promising molecular targeted agent that has been approved for the treatment of unresectable advanced renal cell carcinoma and HCC [7]. However, many patients still develop acquired resistance to sorafenib [8]. It has also recently been reported that sorafenib lacks an anti-HCV effect in HCC patients with HCV [9].

To develop a novel effective therapy for HCV-related HCC with a mechanism of action that is completely different from those of the conventional therapies and established targeted agents, we conducted a comprehensive analysis of the host factors that are specifically overexpressed in HCV-related HCC. We identified 3β-hydroxysterol Δ24-reductase (DHCR24) as a novel host factor that is deeply involved in the pathogenesis of HCV (and the carcinogenesis of hepatic cells) [10]. In HCC cell lines and tissues from patients with IFN-non responsive cirrhosis and HCC, DHCR24 overexpression was regulated at the level of transcription [11]. DHCR24 is also overexpressed in several other cancers [12–16]. DHCR24 is an enzyme that catalyzes the conversion of desmosterol to cholesterol in cholesterol biosynthesis, and it is essential for normal tissue development and maintenance [17,18]. There is a putative transmembrane domain in the N-terminus of DHCR24 [17] and it is primarily localized to the endoplasmic reticulum (ER). In our previous study, we showed that HCV replication can be suppressed by inhibiting DHCR24 with an enzymatic inhibitor, suggesting that DHCR24-mediated cholesterol biosynthesis plays a crucial role in the HCV life cycle [19]. Recently, in a study using a monoclonal antibody against DHCR24 (2-152a MAb), we demonstrated that DHCR24 was specifically expressed on the surface of HCC cell lines [20]. Moreover, high levels of anti-DHCR24 antibodies were detected in the sera of patients with HCV-related HCC (unpublished data). Overexpression of DHCR24 in HCC is specifically induced by HCV [10,21]; therefore, it could be a useful diagnostic marker for HCV-related HCC. Taken together, these findings support a robust correlation between DHCR24 and HCC, especially HCV-related HCC.

Based on our recent findings, we attempted to develop a novel targeting strategy against surface DHCR24 for HCV-related HCC. In this study, we found that 2-152a MAb showed a specific effector function (CDC activity) in HCC cells harboring surface DHCR24. Moreover, independent of the effector function, the 2-152a MAb also possessed agonistic activity against HCV replication, which was mediated by antigen recognition domain binding to surface DHCR24. Taken together, these findings support the feasibility of applying 2-152a MAb to molecular targeted therapy for HCV-related HCC. In addition, we observed that surface DHCR24 could function as a carrier to internalize bound agents into HCC cells. This result suggested the possibility of exploiting this function for specific delivery and incorporation of anticancer drugs into HCC cells. Thus far, abundant cell membrane surface expression of DHCR24 has only been detected on HCC cells. Based on the results of this study, we predict that targeting approach using 2-152a MAb will be an ideal molecular targeted therapy for HCV-related HCC, with both high specificity against cancer cells and anti-HCV activity.

## Materials and Methods

### Cell Lines and Reagents

Human HCC-derived HuH-7 cells were maintained in Dulbecco’s modified Eagle’s medium (DMEM) containing 10% fetal calf serum (FCS; Sigma-Aldrich) with 0.4% glucose. The HuH-7-derived cell line harboring an HCV subgenomic replicon (FLR3-1; genotype 1b) was maintained in DMEM GlutaMAX (Invitrogen) containing 10% FCS in the presence of G418 (500 μg/mL; Invitrogen). The human hepatoblastoma cell line HepG2 was maintained in DMEM containing 10% FCS. PLC/PRF/5 and HEK293 cells were maintained in Eagle’s minimum essential medium (MEM) containing 10% FCS. HuH-7 cells, HepG2 cells, PLC/PRF/5 and HEK293 cells were originally purchased from American Type Culture Collection. U18666A was purchased from Cayman Chemical. Saporin-conjugated anti-mouse IgG (#Mab-ZAP) was purchased from Advanced Targeting Systems.

### Replication Assay using HCV Replicon Cells

We used the HCV subgenomic replicon cell line FLR3-1 (HCV genotype 1b), as described previously [20,22]. FLR3-1 is HuH-7 cells carrying the HCV replicon which is derived from the HCV genotype 1b clone (Gen Bank accession number AY045702) by substituting the neo^r^ gene with the firefly luciferase gene fused to foot-and mouth disease virus (FMDV) 2A and the neo^r^ gene [22]. This modification enables the sensitive quantitation of HCV replication by luciferase assay. Cells were seeded at a density of 5 × 10^3^ cells/well in 96-well tissue culture plates. After incubation for 24 h at 37°C and 5% CO_2_, the medium was removed and serial dilutions of the DHCR24 MAb (2-152a) or derivatives were added. After 72 h, luciferase activity was determined using the Bright-Glo luciferase assay kit (Promega) according to the manufacturer’s instructions. Replication was averaged and is reported relative to the replication of untreated cells, which was set at 100%. The viability of the replicon cells was measured using the WST-8 cell counting kit (Dojindo) according to the manufacturer’s instructions.

### Flow Cytometry to Detect Surface DHCR24

Cell surface expression of DHCR24 protein was assessed by binding studies with 2-152a MAb. In the negative control experiments, normal mouse IgG was used. For flow cytometry analysis, 1 × 10^6^ cells were incubated with 1 g/mL 2-152a MAb, 152a Chimera Ab (ChAb), or 152a scFv-hIgG-Fc at 4°C for 2 h. The cells were then washed 3 times with PBS and incubated with an Alexa Fluor 488-conjugated goat anti-mouse IgG (H+L) antibody or an Alexa Fluor 488-conjugated goat anti-human IgG (H+L) antibody (Molecular Probes) at 4°C for 1 h. The cells were washed again and analyzed using a FACSCalibur flow cytometer (Becton Dickinson). When acquiring data on the flow cytometer, forward scatter (FSC) versus side scatter (SSC) plots were created and it was ensured that all the expected cell populations are visible by adjusting the individual FSC and SSC photomultiplier tube settings. Most of the debris, air bubbles and laser noise (all which should be FSC-low), were removed from analysis by setting and adjusting the FSC threshold. Next, the region around the major cell populations (Region-1: R1) was inserted on the FSC vs SSC plot. The FL1 histogram data was made from the R1 cell population. Data were analyzed using CellQuest software (Becton Dickinson), and the mean fluorescence intensity calculated. Black shades indicate the unstained cell population, the blue line indicate the isotype-reacted cell population and the red line indicate the cell population stained with 2-152a MAb or derivatives.

### Complement-dependent Cytotoxicity Assay

HeLa, HepG2, and HuH-7 cells (1 × 10^6^) were incubated with 1 or 10 μg/mL 2-152a MAb or normal IgG in the presence of guinea pig complement (the complement:cell ratio was 1:8 for HeLa and HepG2 cells and 1:20 for HuH-7 cells) for 30 min at 30°C. After incubation, cell viability was measured using the WST-8 cell counting kit (Dojindo) according to the manufacturer’s instructions.

### Construction of a Chimeric Antibody containing the 2-152a MAb Antigen-binding Domain and the Human IgG Constant Domain

Total RNA was extracted from hybridoma clone #2-152a, and then cDNAs of the variable regions of the light (VL) and heavy (VH) chains were obtained by RT-PCR. The VH and VL cDNAs were inserted into pFUSEss-CHIg-hG1 (InvivoGen) and pFUSE2ss-CLIg-hk (InvivoGen), respectively. These recombinant vectors were co-transfected into HEK293 cells, and then the chimeric antibody was secreted into the culture medium as a soluble protein. The chimeric antibody was purified from the culture medium with protein A/G/L sepharose (BioVision).

### Construction of a scFv Derived from 2-152a MAb and a Fusion Protein with Human IgG1-Fc

Single chain Fv (scFv) fragments derived from 2-152a MAb (VL-VH, VH-VL) were constructed by SOE-PCR and then inserted into pFUSE-hIgG1e4-Fc2 (InvivoGen). These recombinant vectors were transfected into HEK293 cells, and then scFv-human IgG1 Fc fusion proteins were secreted as soluble proteins into the culture medium. The chimeric antibody was purified from the culture medium using protein A/G/L sepharose.

### Western Blot Analysis

Cells (1 × 10^6^) were lysed with 100 μL of lysis buffer (1% SDS, 0.5% Nonidet P-40, 0.15 M NaCl, 0.5 mM EDTA, 1 mM dithiothreitol, and 10 mM Tris, pH 7.4), and then 50 μg of total protein was electrophoresed on an SDS-polyacrylamide gel and transferred to a polyvinylidene difluoride membrane (Immobilon-P; Millipore). The blot was probed with 2-152a MAb to detect DHCR24 protein and with an anti-actin monoclonal Ab (Sigma) to detect actin as an internal loading control.

### Construction of a DHCR24-expressing Plasmid and a Lentiviral Vector

The DHCR24 expression lentiviral vector (rLenti-DHCR24) was constructed as described previously [10]. Briefly, DHCR24 cDNA was cloned into pCSII-EF-MCS-EMCV IRES-Hygro, which was co-transfected into 293FT cells (Invitrogen) with the packaging plasmids pCAG-HIVgp and pCMV-VSVG-RSV-Rev. Following infection, HepG2 cells containing rLenti-DHCR24 were selected with hygromycin B (600 μg/mL; Sigma).

### Cell proliferation ELISA

HuH-7, Hep3B, PLC/PRF/5 and HeLa cells (5 × 10^3^ cells/well in 96-well plates) were treated with 2-152a MAb or mouse IgG (10^–5^–10^1^ μg/mL) in the presence or absence of saporin-conjugated anti-mouse IgG (1 μg/mL). After 72 h, cell viability was then assessed using the BrdU ELISA assay kit (Roche). Average viability was calculated relative to the viability of untreated cells, which was set at 100%.

### Statistical Analysis

Student’s *t*-test was used to analyze the statistical significance of the results. *P* values less than 0.05 were considered statistically significant.

## Results

### Expression of DHCR24 on the Cell Surface of HCC Cell Lines

**Fig 1:**
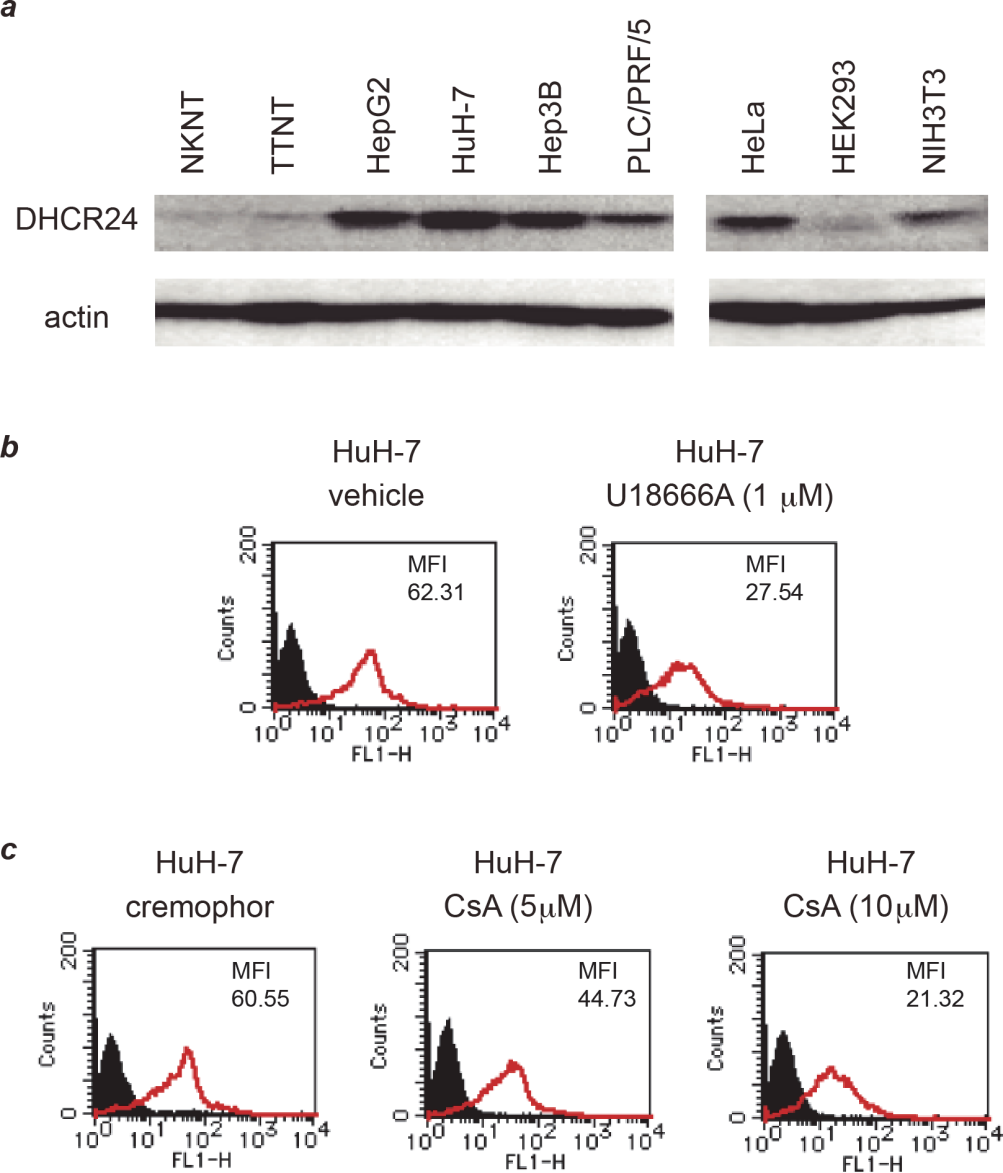
Overexpression of DHCR24 on the cell surface of HCC cell lines was decreased by treatment with U18666A and cyclosporin A. (*a*) Cell lysates of each cell line (containing 50 μg of protein) were separated by 10% SDS-PAGE and analyzed by western blotting with 2-152a MAb and an anti-actin MAb. Normal hepatic cell lines: NKNT, and TTNT. HB-derived cell line: HepG2. HCC-derived cell lines: HuH-7, Hep3B, and PLC/PRF/5. (*b*) HuH-7 cells were treated with U18666A (final concentration, 1 μM) for 48 h, and then the surface expression of DHCR24 was analyzed by flow cytometry. (*c*) HuH-7 cells were treated with cyclosporin A (final concentration, 5 or 10 μM) or solvent (cremophor) for 48 h, and then the surface expression of DHCR24 was analyzed by flow cytometry.

Overexpression of DHCR24 was observed in HCC and hepatoblastoma (HB) cell lines but not in hepatic cell lines derived from normal liver tissue (Fig 1*A*). Using a monoclonal antibody against DHCR24 (2-152a MAb), we detected DHCR24 expression on the cell surface of HCC cell lines (HuH-7, Hep3B, etc.) and the HB cell line HepG2 [20]. In contrast, no DHCR24 expression was detected on the surface of the normal human hepatocyte lines NKNT and TTNT, which also express low levels of intracellular DHCR24 protein (Fig 1*A*, S1 Fig, and S1 Table). Surface expression of DHCR24 was also not detected in the cervical adenocarcinoma-derived cell line HeLa, even when intracellular DHCR24 protein was overexpressed (Fig 1*A* and S1 Fig). These findings suggest that the expression of DHCR24 on the cell surface is a unique property of HCC and HB cells.

DHCR24 catalyzes the conversion of desmosterol to cholesterol in the cholesterol biosynthetic pathway [17,18]. Next, we examined the relationship between DHCR24 enzymatic activity and its surface expression. Incubation of HuH-7 cells with U18666A, an inhibitor of both DHCR24 and intracellular cholesterol transport, significantly decreased DHCR24 surface expression (Fig 1*B*). This result suggested that the enzymatic activity of DHCR24 and/or the cholesterol transport machinery are involved in DHCR24 surface expression. In addition, cyclosporin A (CsA) treatment also decreased the DHCR24 surface expression in a dose-dependent manner (Fig 1*C* and S2 Fig). CsA is an inhibitor of cyclophilin, which has been reported to be part of a cytosolic trafficking complex consisting of caveolin, heat-shock protein 56, cyclophilin 40, cyclophilin A, and cholesterol [23]. CsA treatment disrupted this transport complex, which prevented cholesterol transport to the caveolae at the plasma membrane (PM) [23]. Taken together, these findings suggested that surface expression of DHCR24 in HCC cells is associated with cholesterol transport.

### Identification of the DHCR24 Recognition Site in 2-152a MAb and Generation of a Chimeric Antibody

**Fig 2:**
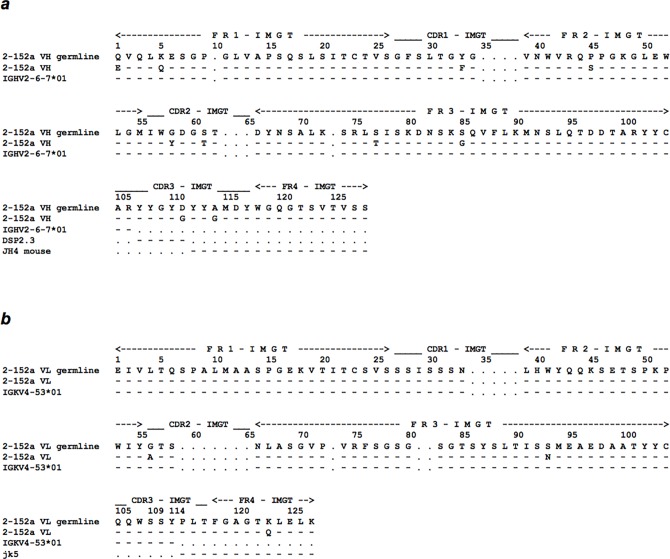
Identification of the surface DHCR24-recognition domain of 2-152a MAb. (*a*) Alignment of the VH amino acid sequence of 2-152a MAb with the germline configuration. The V, D, J, and CDR sequences were determined by using the IMGT database (http://www.imgt.org/). (*b*) Alignment of the VL amino acid sequence of 2-152a MAb with the germline configuration. The V, J, and CDR sequences were determined by using the IMGT database.

Previously, we showed that the 2-152a MAb possessed agonistic activity against HCV replication independent of the effector function [20]. We hypothesized that the antigen recognition domain of 2-152a MAb is essential for the agonistic activity against surface DHCR24. To test this hypothesis, we first identified the antigen recognition site (variable region) in the light (VL) and heavy (VH) chains of 2-152a MAb (Fig 2). V, D, and J sequence analysis using the IMGT database showed that 2-152a MAb utilizes IGHV2-6-7*01 and IGKV4-53*01 and carries 10 somatic mutations in VH and 3 in VL (Fig 2*A* and 2*B*).

**Fig 3:**
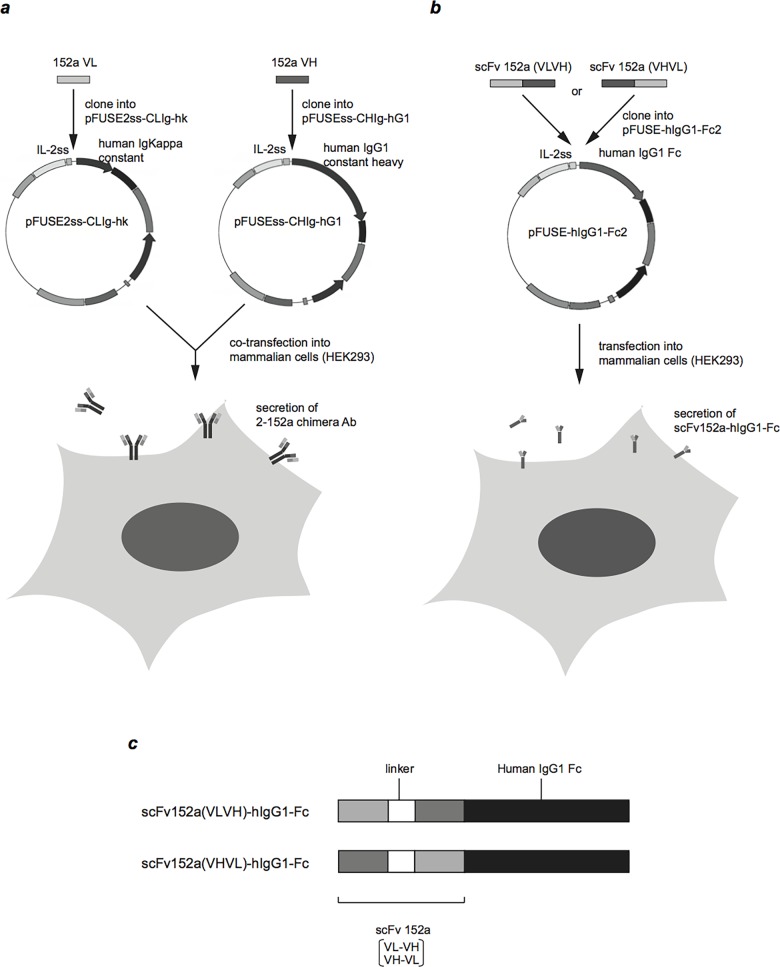
Construction of a chimeric antibody and scFv consisting of the 2-152a MAb antigen-binding domain and the human IgG constant domain. (*a*) The 2-152a VL and VH cDNAs were isolated from hybridoma #2-152a and cloned into pFUSE2ss-CLIg-hk and pFUSEss-CHIg-hG1, respectively. HEK293 cells were co-transfected with the expression vector harboring the chimeric Ig (152a Ch-L; pFUSE2ss-152aVL-CLIg-hk, 152a Ch-H; pFUSEss-152aVH-CHIg-hG1). The 152a Chimera Ab or chimeric Ig (152a Ch-L, 152a Ch-H) was secreted into the culture medium and then purified from the culture medium by using protein A/G/L sepharose. (*b*) The 152a scFvfragments (152a VL-VH, 152a VH-VL) were constructed by SOE-PCR and cloned into pFUSE-hIgG1e4-Fc2. HEK293 cells were transfected with the 152a scFv-hIgG1-Fc expression vectors (pFUSE-scFv152a(VLVH)-hIgG1-Fc and pFUSE-scFv152a(VHVL)-hIgG1-Fc). 152a scFv-hIgG1-Fc fusion protein was secreted into the culture medium, and then the protein was purified from the culture medium by using protein A/G/L sepharose. (*c*) Schematic diagram of scFv152a-hIgG1-Fc. The scFv fragments (152a VL-VH or 152a VH-VL) derived from 2-152a MAb were fused to the Fc portion of human IgG1.

**Fig 4:**
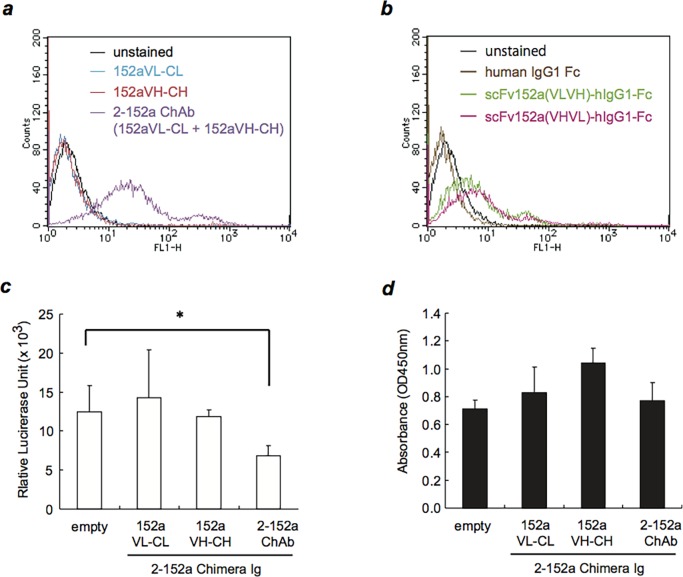
152a ChAb can bind to surface DHCR24 and shows anti-HCV activity. (*a*) HuH-7 (1 × 10^6^) cells were incubated with the light or heavy chain of the chimeric Ig (152a Ch-L, 152a Ch-H) or 152a ChAb (1 μg/mL, respectively) at 4°C for 2 h, and then with an Alexa Fluor 488-conjugated goat anti-human IgG at 4°C for 1 h. The cells were then analyzed by flow cytometry. (*b*) HuH-7 (1 × 10^6^) cells were incubated with 152a scFv-hIgG1-Fc at 4°C for 2 h, and then incubated with Alexa Fluor 488-conjugated goat anti-human IgG at 4°C for 1 h. The cells were then analyzed by flow cytometry. (*c*) Subgenomic HCV replicon FLR3-1 cells were plated in a 96-well plate at a density of 5 × 10^3^ cells/well and allowed to adhere overnight. Then, the supernatant was removed, and the cells were treated with the light or heavy chain of the chimeric Ig (152a Ch-L, 152a Ch-H) or 152a ChAb (1 μg/mL, respectively) for 72 h. HCV replication was evaluated by measuring luciferase activity using the Bright-Glo Luciferase Assay System. *, *p* < 0.05. (*d*) Simultaneously, the viability of FLR3-1 cells was evaluated by measuring the absorbance (OD at 450 nm) using the WST-8 Cell Counting Kit. Experiments were performed 3 times with triplicate wells.

Next, we constructed a chimeric antibody consisting of the VL and VH of 2-152a MAb fused to the constant regions of human Ig kappa and Ig gamma-1 (Fig 3*A*), respectively, which was designated as 152a Chimera Ab (ChAb). As shown in Fig 4*A*, similar to 2-152a MAb, 152a ChAb could detect surface DHCR24 on HuH-7 cells. We also constructed two scFvs derived from 2-152a MAb (152a VL-VH and 152a VH-VL). We fused these scFvs to the Fc portion of human IgG1, and designated them as 152a scFv(VLVH)-hIgG1-Fc and 152a scFv(VHVL)-hIgG1-Fc (Fig 3*B* and 3*C*). Both of the 152a scFv-hIgG1-Fc fusion proteins could recognize surface DHCR24 on HuH-7 cells (Fig 4*B*); however, the binding potential was lower than that of 152a ChAb (Fig 4*A*). These data suggest that 152a ChAb is more suitable rather than 152a scFv-hIgG1-Fc as a delivery tool for HCC. Furthermore, we examined whether 152a ChAb possessed anti-HCV activity similar to that of 2-152a MAb. Treatment with 152a ChAb significantly suppressed HCV replication in HCV replicon cells (Fig 4*C*) independent of cytotoxicity (Fig 4*D*). In contrast, treatment with the heavy or light chain of 152a ChAb alone (152a Ch-L or 152a Ch-H) did not suppress HCV replication (Fig 4*C*) and could not detect surface DHCR24 (Fig 4*A*). These results suggest that binding of 2-152a MAb to surface DHCR24, which is mediated through the antigen-binding domain, is necessary and sufficient for its anti-HCV activity.

### Antitumor Activity of 2-152a MAb mediated through its Effector Function

**Fig 5:**
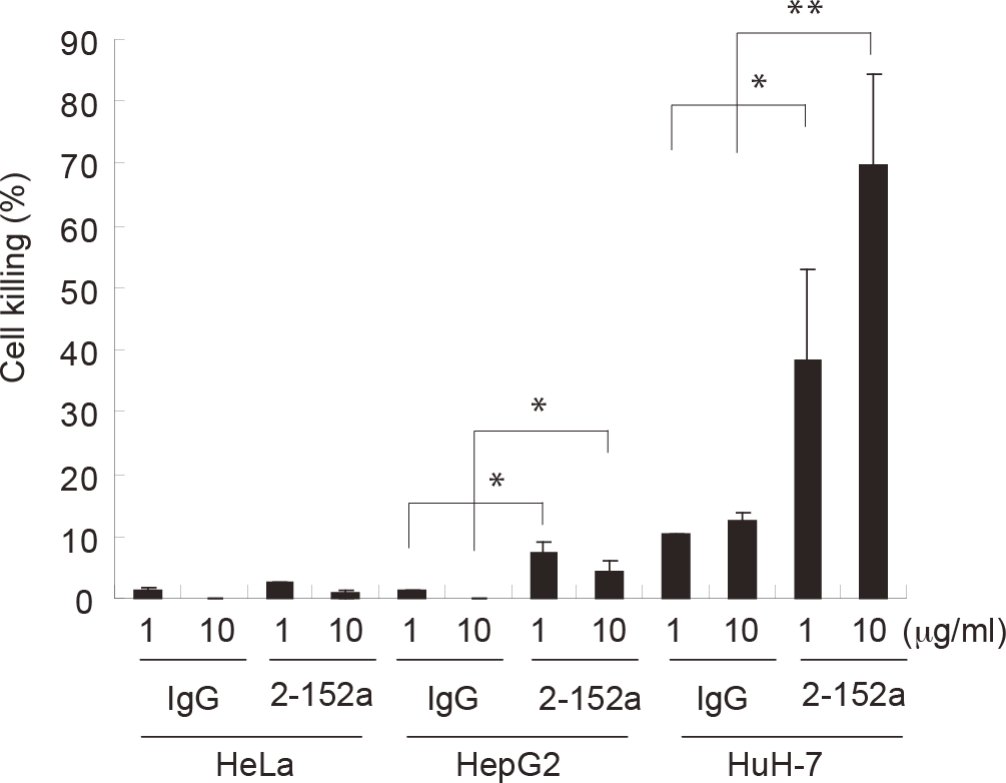
Complement-dependent cytotoxicity (CDC) induced by 2-152a MAb. HeLa, HepG2, and HuH-7 cells (1 × 10^6^) were incubated with 2-152a MAb or mouse IgG (final concentration, 1 or 10 μg/mL) in the presence of guinea pig complement (the complement:cell ratio was 1:8 for HeLa and HepG2 and 1:20 for HuH-7) for 30 minutes at 30°C. Cell viability was measured using the WST-8 cell counting kit, and cell killing was calculated by comparing death in the experimentally treated cells with that in the untreated cells. *, *p* < 0.05; **, *p* < 0.01.

To examine the feasibility of using 2-152a MAb for molecular targeted therapy against surface DHCR24 in HCC, we examined the effector function of 2-152a MAb for antitumor activity. In the presence of complement, 2-152a MAb showed cytotoxicity against HuH-7 cells in a dose-dependent manner, which was mediated through complement-dependent cytotoxicity (CDC; Fig 5). Significant CDC activity was also observed in HepG2 cells, which only express modest levels of surface DHCR24. In contrast, no CDC activity was observed in HeLa cells. These results suggest that 2-152a MAb possesses the effector function that is fundamental for antibody therapy and it is correlated with the surface expression of DHCR24. These findings indicate that 2-152a MAb could be used for antibody therapy against HCC.

### DHCR24 on the Surface of HCC Cells Can Function as Carrier of Targeted Agents

**Fig 6:**
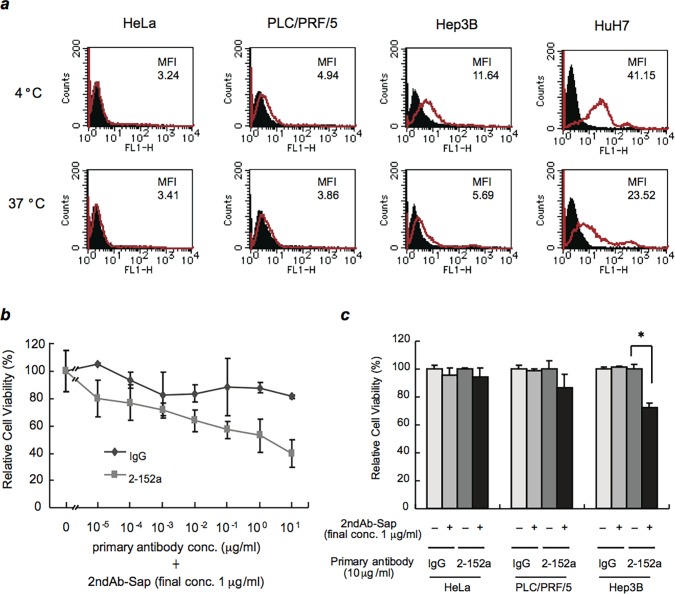
Specific uptake mediated by cell-surface DHCR24. (*a*) HCC cell lines (HuH-7, Hep3B, and PLC/PRF/5) and HeLa cells were incubated with 2-152a MAb at 4°C (a temperature that inhibits endocytosis) or 37°C (physiological temperature) for 2 h, and then incubated with an Alexa Fluor 488-conjugated goat anti-mouse IgG at 4°C for 1 h. The cells were then analyzed by flow cytometry. (*b*) HuH-7 cells were seeded at a density of 5 × 10^3^ cells/well in 96-well tissue culture plates. After incubation for 24 h, serial dilutions of 2-152a MAb or mouse IgG were added in the presence of saporin-conjugated anti-mouse IgG (1 μg/mL). After 72 h, cell viability was then assessed using the BrdU ELISA assay kit. Average viability was calculated relative to the viability of untreated cells, which was set at 100%. (*c*) HeLa, Hep3B, and PLC/PRF/5 cells were treated with 2-152a MAb or mouse IgG (10 μg/mL) in the presence or absence of saporin-conjugated anti-mouse IgG (1 μg/mL). After 72 h, cell viability was determined using a BrdU ELISA assay kit. Percent viability was calculated relative to the viability of untreated cells, which was set at 100%. *, *p* < 0.05.

In response to 2-152a MAb binding, the expression of surface DHCR24 in HCC cell lines was downregulated (Fig 6*A* and S3 Fig). In contrast, surface expression of DHCR24 in HB-derived HepG2 cells was not downregulated in response to 2-152a MAb binding (S4 Fig). Based on these findings, we postulated that surface DHCR24 on HCC cells might function as a carrier to transport bound agents into cells. Therefore, we examined whether surface DHCR24 could be a valuable target for the delivery of anticancer drugs to HCC. HCC cell lines were treated with 2-152a MAb in combination with a saporin-conjugated secondary antibody (2ndAb-Sap). Saporin, a ribosome-inactivating protein, enzymatically inactivates ribosomes, shutting down protein synthesis and causing cell death. Therefore, 2-152a MAb-mediated binding of 2ndAb-Sap to surface DHCR24 on HCC cells was predicted to lead to internalization of 2ndAb-Sap and subsequent cell death. As shown in Fig 6*B*, in the presence of 2ndAb-Sap, 2-152a MAb showed a significant, dose-dependent cytotoxic effect on HuH-7 cells, whereas control mIgG combined with 2ndAb-Sap showed little cytotoxicity. These results suggest that the cytotoxic effect of 2ndAb-Sap could be mediated by 2-152a MAb. Furthermore, in other HCC cell lines, a cytotoxic effect of 2ndAb-Sap mediated by 2-152a MAb was also observed (Fig 6*C*), which correlated with the expression level of surface DHCR24 (Fig 6*A* and S1 Table). Additionally, similar to the lack of CDC activity of 2-152a MAb observed in surface DHCR24-negative HeLa cells (Fig *4D*), there was no cytotoxic effect of 2ndAb-Sap in HeLa cells. Taken together, these results indicate that 2ndAb-Sap was internalized in response to the binding of 2-152a MAb to surface DHCR24 on HCC cells, which led to the cytotoxic effect. In other words, cell surface DHCR24 might function as a carrier to internalize bound cytotoxic agents into HCC cells. These findings show a novel function for surface DHCR24 in HCC, and provide evidence for the feasibility of exploiting this function for the delivery and incorporation of anticancer drugs into HCC cells.

## Discussion

Building on the findings of our recent studies [10,11,19–21], here, we aimed to exploit the surface expression of DHCR24 to develop a novel molecular targeted strategy for HCV-related HCC. In HCC cell lines, DHCR24 was overexpressed not only intracellularly but also on the cell surface [20] (Fig 1*A* and S1 Table). We discovered that DHCR24 surface expression on HCC-derived HuH-7 cells was significantly suppressed by both U18666A, an inhibitor of DHCR24 and cholesterol transport (Fig 1*B*), and CsA, an inhibitor of cyclophilin (Fig 1*C*). In the cell, DHCR24 is primarily localized to the ER, and it converts desmosterol to cholesterol in the cholesterol biosynthetic pathway [17,18]. Cholesterol is synthesized in the ER and is transported to other organelles and the PM through a combination of vesicular and non-vesicular transport processes [24–28]. Non-vesicular transport of cholesterol from the ER to the PM proceeds via a cytosolic trafficking complex consisting of caveolin, hsp56, cyclophilin 40, cyclophilin A, and cholesterol [23]. Based on this information and our results, we thought that DHCR24 is transported from the ER to the PM in association with cholesterol, or that DHCR24 itself might function as a carrier of cholesterol. Additionally, the results of previous studies have suggested that cellular cholesterol content influences the expression of surface molecules on the PM. For example, surface expression of CD81 on HuH-7 cells was decreased after cholesterol depletion [29]. Therefore, the enzymatic activity of DHCR24 in cholesterol synthesis might also affect its surface expression (Fig 1*B*). Cyclophilin also has peptidyl-prolyl cis-trans isomerase (foldase) and molecular chaperone activities. The CsA-mediated post-translational reduction of DHCR24 surface expression might result from inhibition of these cyclophilin activities, which are also required for cell surface externalization of the insulin receptor [30] and creatine transporter [31]. Furthermore, cyclophilin overexpression has been recently reported in diverse types of cancer, including HCC [32,33]. Taken together, cyclophilins might be the important factors for abundant surface expression of DHCR24 on HCC-derived cells.

Our most intriguing finding is the anti-HCV activity of anti-DHCR24 monoclonal antibody (2-152a MAb) [20]. This anti-HCV activity was not accompanied by cytotoxicity, suggesting that it is a unique function of 2-152a MAb, independent of the effector function, which is a basic function of therapeutic antibody. In the current study, we found that 152a ChAb also possessed anti-HCV activity which was similar to that of 2-152a MAb (Fig 4*C*), suggesting that the Fv portion of 2-152a MAb is essential for this function. Our data strongly suggest that surface DHCR24 is involved in the 2-152a MAb/152a ChAb-mediated anti-HCV activity. Therefore, the role of surface DHCR24 in the HCV life cycle should also be analyzed.

In this study, the effector function of 2-152a MAb was demonstrated by CDC activity against HCC cells (Fig 5), which suggests the possibility of using 2-152a MAb for antibody therapy against HCC. Taken together with the above-mentioned anti-HCV activity, these findings lend support to the feasibility of using 2-152a MAb in molecular targeted therapy for HCC, especially HCV-related HCC. However, 2-152a MAb is a mouse IgG1, which are generally thought to possess lower Antibody-dependent cell-mediated cytotoxicity (ADCC) and CDC activity than other mouse IgG isotypes, and the Fc portion of 2-152a MAb is different from that of human IgG1. Therefore, 2-152a MAb might not exert adequate effector function (ADCC and CDC) in humans. In addition, in humans, antibodies would likely be raised against this mouse IgG following administration of this xenogeneic antibody. The chimeric antibody (152a ChAb) developed in this study might address these issues. 152a ChAb consists of the variable region of 2-152a MAb and the constant region of human IgG1 (Fig 3*A*). The functions and properties of 152a ChAb were similar to those of 2-152a MAb (Fig 4*A* and 4*C*). Therefore, 152a ChAb might be able to overcome the above-mentioned issues with 2-152a MAb. We expect that 152a ChAb possesses a more potent effector function due to substitution of the mouse IgG1 Fc portion with that of human IgG1, which shows higher ADCC and CDC activity in humans. Taken together, construction of 152a ChAb enhances the feasibility of using an anti-DHCR24 MAb as a molecular targeted agent against HCV-related HCC in the future.

Moreover, we found that cell surface DHCR24 could function as a carrier to internalize bound agents into HCC cells (Fig 6), suggesting that surface DHCR24 could be used for molecular targeted therapy and antibody therapy against HCC. At present, abundant surface expression of DHCR24 has only been observed in HCC cells [20] (S1 Table). Before surface DHCR24 can be used in clinical applications as a specific target in HCC, it is necessary to verify the surface expression of DHCR24 in primary hepatic cells and liver tissue derived from HCC and to compare the surface expression levels of DHCR24 in HCC cells, normal hepatocytes, and other types of cancer cells. In HB-derived HepG2 cells, surface expression of DHCR24 was slightly induced by 2-152a MAb treatment (S4 Fig). Therefore, in some types of cancer cells, surface expression of DHCR24 might be induced by certain stimuli. The molecular mechanism underlying the constitutive surface expression of DHCR24 in HCC cells is not yet known. Therefore, we should elucidate the entire mechanism before applying surface DHCR24 for clinical use as a therapeutic target against HCC.

HCC does not respond to most chemotherapeutic drugs, so there is an urgent need to develop new drugs with different mechanism of action. Targeted therapy using MAb represents a novel promising strategy. Actually, some clinical trials of antibody therapies for advanced HCC are in progress [34–37]. Recently, novel HCC-specific target molecules such as Glypican-3 (GPC3), EGFR variant III (EGFRvIII), granulin-epithelin precursor (GEP) and AF-20 antigen were discovered and attempts to treat HCC by using the therapeutic antibody targeting these molecules are underway [38–46]. There are mainly two patterns on the usage of the targeting antibody. One usage is to utilize the effector function of the antibody (ADCC, CDC) and antagonistic action that inhibit proliferative signaling pathway in cancer. Anti-GPC3 MAb and anti-EGFRvIII MAb show antitumor activity mediated through ADCC and/or CDC [43,45,46]. In addition, anti-EGFRvIII MAb, anti-GEP MAb and VH domain antibody derived from anti-GPC3 MAb inhibit EGFR signaling, GEP signaling and Hippo signaling, respectively [40,43,44], which are strongly activated in HCC. Additively, these therapeutic antibodies could sensitize HCC to the chemotherapeutic agents [41,42,46]. Another usage is to utilize the antibody as delivery tool for cancer and incorporate the cytotoxic payload to cancer cells mediated through the bound antibody. Binding of AF-20 MAb to AF-20 antigen on the cell surface of HCC triggers rapid internalization at 37°C [38,39]. Based on this property, targeting approaches for HCC such as the transfer of suicide genes and incorporation of chemotherapeutic agents are devised by using AF-20 MAb [38,39]. 2-152a MAb could fulfill these both usages (Fig 5 and Fig 6), therefore, can be a promising therapeutic antibody against HCC.

## Supporting Information

S1 Fig Expression of surface DHCR24 in NKNT, TTNT and HeLa cells. NKNT, TTNT and HeLa cells (1 x 10^6^) were incubated with 1 μg/mL 2-152a MAb at 4°C for 2 h, and then incubated with Alexa Fluor 488-conjugated goat anti-mouse IgG at 4°C for 1 h. The cells were then analyzed by flow cytometry. Black shades indicate the unstained cell population and the red line indicate the stained cell population.
(TIF)

S2 Fig The surface expression of DHCR24 in HCV replicon cells was decreased by treatment with cyclosporin A. R6FLR-N cells were treated with cyclosporin A (final concentration, 5 or 10 μM) or solvent (cremophor) for 48 h, and then the surface expression of DHCR24 was analyzed by flow cytometry. Black shades indicate the unstained cell population and the red line indicate the stained cell population.
(TIF)

S3 Fig The surface expression of DHCR24 in HCV replicon cells was downregulated by binding of 2-152a MAb. HCV replicon cell lines (R6FLR-N, FLR3-1 and Rep-JFH) and cured HuH-7/K4 cells were incubated with 2-152a MAb at 4°C (a temperature that inhibits endocytosis) or 37°C (physiological temperature) for 2 h, and then incubated with Alexa Fluor 488-conjugated goat anti-mouse IgG at 4°C for 1 h. The cells were then analyzed by flow cytometry. Black shades indicate the unstained cell population, the blue line indicate the isotype-reacted cell population and the red line indicate the stained cell population.
(TIF)

S4 Fig The surface expression of DHCR24 in an HB-derived cell line was not internalized in response to the binding of 2-152a MAb. HepG2 and HepG2 infected with a DHCR24 lentiviral vector (rLenti-DHCR24) or an empty vector (rLenti-empty) were incubated with 2-152a MAb at 4°C (a temperature that inhibits endocytosis) or 37°C (physiological temperature) for 2 h, and then incubated with Alexa Fluor 488-conjugated goat anti-mouse IgG at 4°C for 1 h. The cells were then analyzed by flow cytometry. Black shades indicate the unstained cell population and the red line indicate the stained cell population.
(TIF)

S1 Table Intracellular and cell surface expression of DHCR24 (DOC)
