# Supplementary figures and images for: 3β-Hydroxysterol Δ24-Reductase on the Surface of Hepatitis C Virus-Related Hepatocellular Carcinoma Cells Can Be a Target for Molecular Targeting Therapy
Source: PLoS One. 2015 Apr 13;10(4):e0124197. doi: 10.1371/journal.pone.0124197 (PMC4395381; doi:10.1371/journal.pone.0124197)

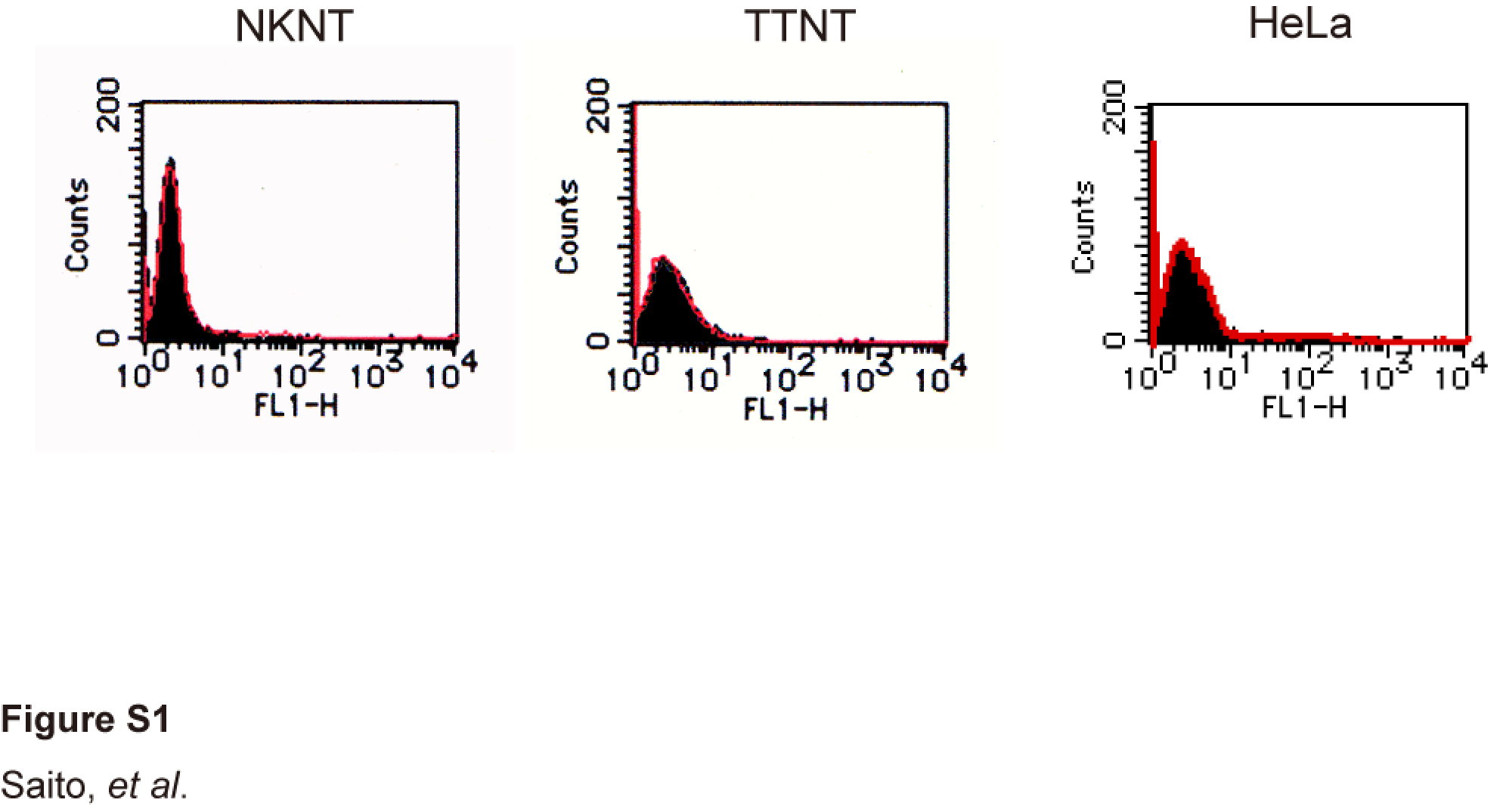

Supplement: S1 Fig — NKNT, TTNT and HeLa cells (1 x 10^6^) were incubated with 1 μg/mL 2-152a MAb at 4°C for 2 h, and then incubated with Alexa Fluor 488-conjugated goat anti-mouse IgG at 4°C for 1 h. The cells were then analyzed by flow cytometry. Black shades indicate the unstained cell population and the red line indicate the stained cell population. (TIF) [file pone.0124197.s001.tif]

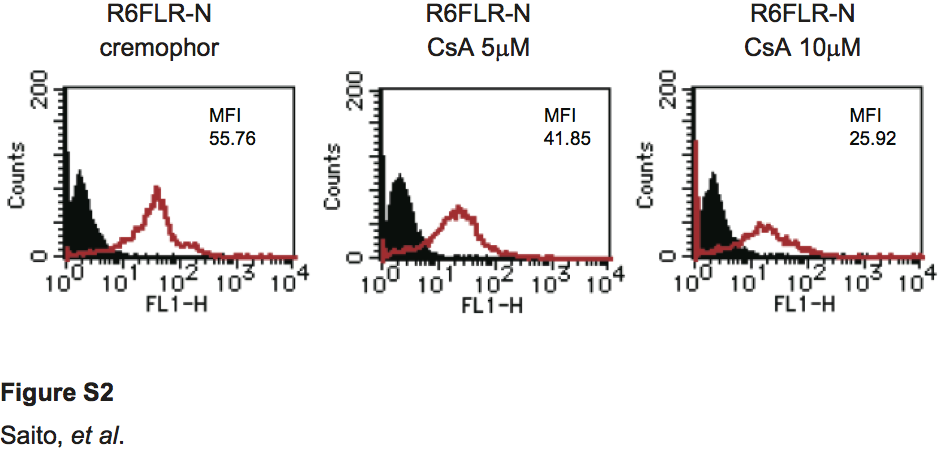

Supplement: S2 Fig — R6FLR-N cells were treated with cyclosporin A (final concentration, 5 or 10 μM) or solvent (cremophor) for 48 h, and then the surface expression of DHCR24 was analyzed by flow cytometry. Black shades indicate the unstained cell population and the red line indicate the stained cell population. (TIF) [file pone.0124197.s002.tif]

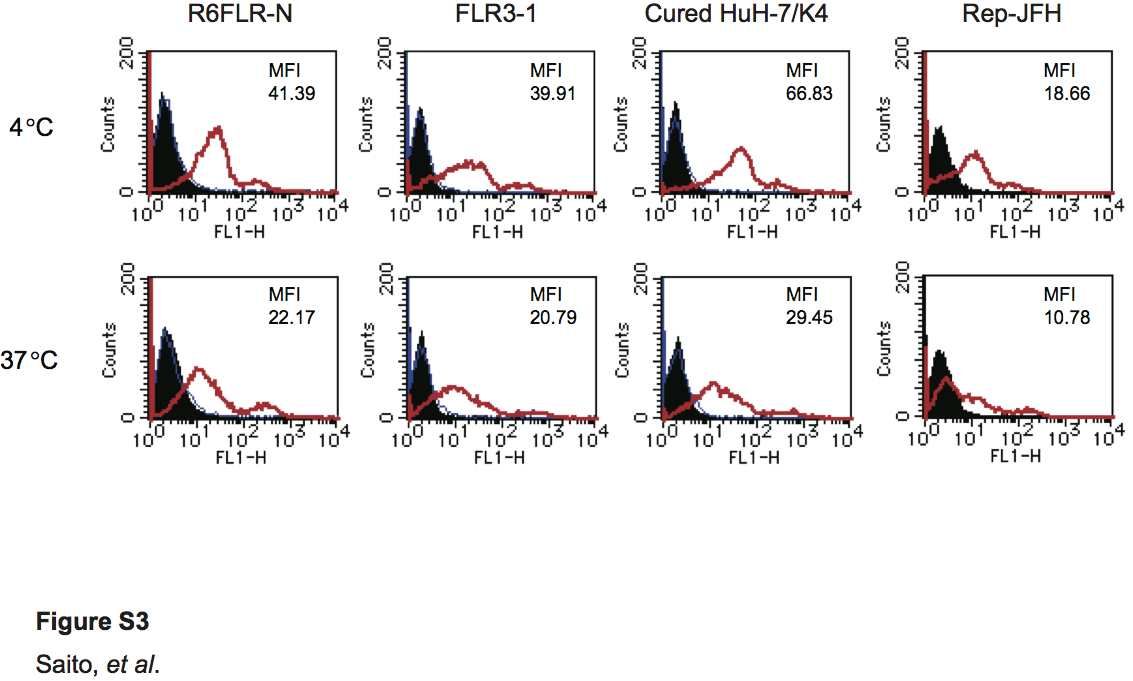

Supplement: S3 Fig — HCV replicon cell lines (R6FLR-N, FLR3-1 and Rep-JFH) and cured HuH-7/K4 cells were incubated with 2-152a MAb at 4°C (a temperature that inhibits endocytosis) or 37°C (physiological temperature) for 2 h, and then incubated with Alexa Fluor 488-conjugated goat anti-mouse IgG at 4°C for 1 h. The cells were then analyzed by flow cytometry. Black shades indicate the unstained cell population, the blue line indicate the isotype-reacted cell population and the red line indicate the stained cell population. (TIF) [file pone.0124197.s003.tif]

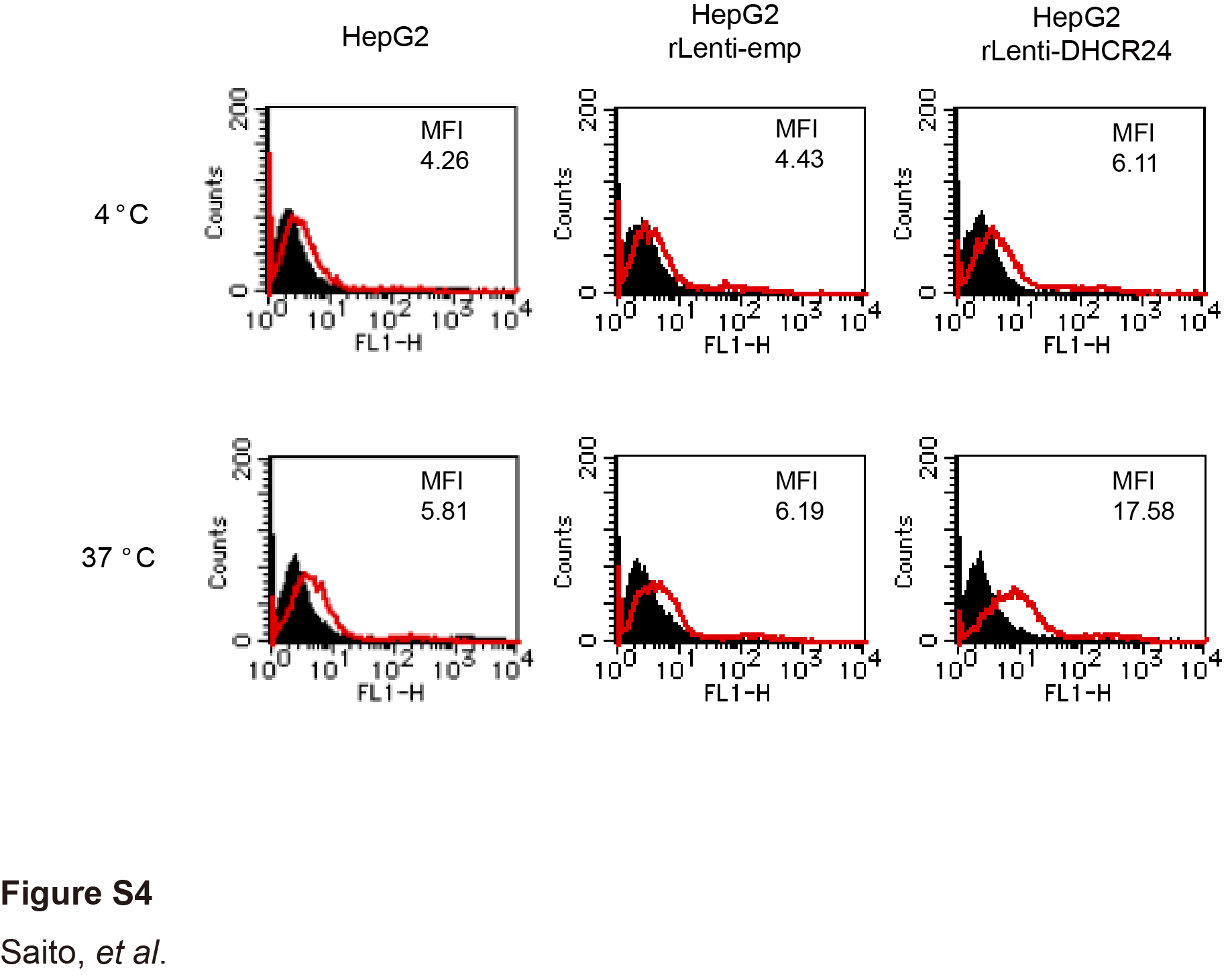

Supplement: S4 Fig — HepG2 and HepG2 infected with a DHCR24 lentiviral vector (rLenti-DHCR24) or an empty vector (rLenti-empty) were incubated with 2-152a MAb at 4°C (a temperature that inhibits endocytosis) or 37°C (physiological temperature) for 2 h, and then incubated with Alexa Fluor 488-conjugated goat anti-mouse IgG at 4°C for 1 h. The cells were then analyzed by flow cytometry. Black shades indicate the unstained cell population and the red line indicate the stained cell population. (TIF) [file pone.0124197.s004.tif]
