# Supplementary material for: 3β-Hydroxysterol Δ24-Reductase on the Surface of Hepatitis C Virus-Related Hepatocellular Carcinoma Cells Can Be a Target for Molecular Targeting Therapy
Source: PLoS One. 2015 Apr 13;10(4):e0124197. doi: 10.1371/journal.pone.0124197 (PMC4395381; doi:10.1371/journal.pone.0124197)
Supplement: S1 Table — (DOC) [file pone.0124197.s005.doc]

**S1 Table**. Intracellular and cell surface expression of DHCR24

| Cell line | Intracellular  DHCR24 | Surface  DHCR24 | HCV | Origin |
| --- | --- | --- | --- | --- |
| HuH-7 | ++ | ++ | - | HCC |
| R6FLR-N | ++ | ++ | + (sub) | HCC |
| FLR3-1 | ++ | ++ | + (sub) | HCC |
| Rep-JFH | ++ | ++ | + (sub) | HCC |
| JFH/K4 | ++ | ++ | + (full) | HCC |
| Cured HuH-7/K4 | ++ | ++ | - | HCC |
| Hep3B | + | + | - | HCC (HBV+) |
| PLC/PRF/5 | + | + | - | HCC |
| HepG2 | + | ± | - | HB |
| HepG2/rLenti-emp | + | ± | - | HB |
| HepG2/rLenti-DHCR24 | ++ | + | - | HB |
| TTNT | - | - | - | NH |
| NKNT | - | - | - | NH |
| HeLa | + | - | - | CA |

HCC, hepatocellular carcinoma; HB, hepatoblastoma; NH, normal hepatic tissue; CA, cervical adenocarcinoma.
